# Bridging the gap between radiologic and manometric criteria to diagnose esophageal motility disorders: a pictorial review for radiologists

**DOI:** 10.1093/bjro/tzag005

**Published:** 2026-03-01

**Authors:** Ahmed O El Sadaney, D Chamil Codipilly, David J Bartlett, Kristina T Flicek, Michael L Wells, Safa Hoodeshenas, Jay Heiken, Karthik Ravi, Joel G Fletcher, Avinash K Nehra

**Affiliations:** Department of Radiology, Mayo Clinic, Rochester, MN 55905, United States; Department of Gastroenterology, Mayo Clinic, Rochester, MN 55905, United States; Department of Radiology, Mayo Clinic, Rochester, MN 55905, United States; Department of Radiology, Mayo Clinic, Rochester, MN 55905, United States; Department of Radiology, Mayo Clinic, Rochester, MN 55905, United States; Department of Radiology, Mayo Clinic, Rochester, MN 55905, United States; Department of Radiology, Mayo Clinic, Rochester, MN 55905, United States; Department of Gastroenterology, Mayo Clinic, Rochester, MN 55905, United States; Department of Radiology, Mayo Clinic, Rochester, MN 55905, United States; Department of Radiology, Mayo Clinic, Rochester, MN 55905, United States

**Keywords:** esophageal motility disorders, esophagus, manometry, fluoroscopy, lower esophageal sphincter

## Abstract

Dysphagia is defined as a subjective sensation of difficulty swallowing and can result from oropharyngeal or esophageal etiologies based upon patient symptoms. Dysphagia affects approximately 16% of adults in the general population, with prevalence increasing with age. Esophagogastroduodenuoscopy (EGD) is initially performed to assess for structural abnormalities resulting in esophageal dysphagia. However, if EGD reveals no pathologic abnormalities, high-resolution manometry (HRM) and barium esophagography are performed in order to assess for underlying causes of dysmotility. Esophageal motility disorders (EMDs) are an underrecognized cause of dysphagia and can be characterized by impaired esophageal peristalsis or lower esophageal sphincter dysfunction. High-resolution manometry (HRM) measures key metrics such as integrated relaxation pressure (IRP), which is the deglutitive relaxation across the LES, and metrics of esophageal body peristalsis based on distal contractile integral (DCI) and distal latency (DL). The Chicago Classification version 4 (CCv4.0), published in 2021, provides a standardized classification scheme for differentiating EMDs using metrics from HRM. Additionally, barium esophagography has remained an important adjunctive diagnostic modality, as this may identify strictures, neoplasms, or hiatal hernias, but can also identify major motility disorders such as achalasia and distal esophageal spasm. The combined use of HRM with timed barium esophagram can enhance the diagnostic accuracy of EMDs, particularly when HRM demonstrates inconclusive findings. Therefore, radiologists should be familiar with how imaging findings from barium esophagram integrate with findings noted on HRM. The aim of this review is to highlight the findings of EMDs noted on HRM in conjunction with barium esophagography, thereby illustrating characteristic patterns of primary and secondary EMDs.

## Introduction

Dysphagia is defined as the subjective sensation of difficulty swallowing and is a relatively common and increasingly prevalent clinical problem. Population-based studies report that dysphagia affects approximately 16% of adults.^1^ Dysphagia can be further classified as oropharyngeal or esophageal in etiology, depending upon the location of symptoms. Esophagogastroduodenuoscopy (EGD) is initially performed to assess for abnormalities that may result in esophageal dysphagia; however, in the absence of benign or malignant abnormalities, high-resolution manometry (HRM) and barium esophagram are useful in order to assess for underlying causes of dysmotility.

Esophageal motility disorders (EMDs) may also result in dysphagia and include diagnoses such as achalasia, esophagogastric junction outflow obstruction (EGJOO), absent contractility, distal esophageal spasm (DES), hypercontractile esophagus, and ineffective esophageal motility (IEM). Patients with EMDs typically present with dysphagia to both solids and liquids. In contrast, patients with structural causes of dysphagia characteristically present with dysphagia to solids, which may progress to also include liquids. Additional symptoms may include heartburn, regurgitation, and non-cardiac chest pain. Esophageal motility disorders may be subcategorized into primary and secondary disorders. Primary EMDs are those that are primarily related to the esophagus due to inadequate relaxation of the lower esophageal sphincter or impaired esophageal peristalsis.^2^ Secondary EMDs are due to a systemic disease with esophageal involvement, including scleroderma, Chagas disease, or post-surgical esophageal dysfunction.^3^

The aim of this review is to highlight the findings of EMDs noted on HRM as interpreted using the Chicago Classification version 4.0 (CCv4.0) and to demonstrate how HRM in conjunction with barium esophagography can illustrate characteristic patterns of primary and secondary EMDs.

### High-resolution esophageal manometry

High-resolution manometry is the gold standard test in the assessment of EMDs. The Chicago Classification is the most widely used classification system for evaluation, and diagnosis of EMDs using metrics from esophageal HRM, although endoscopic or radiographic evidence of mechanical obstruction precludes the use of CCv4.0. The most recent CCv4.0 was published in 2021 to update diagnostic criteria standardize the protocol for evaluation, including both supine and upright test positions as well as the use of provocative testing (multiple rapid swallows and rapid drink challenge).^4^

**Figure 1:**
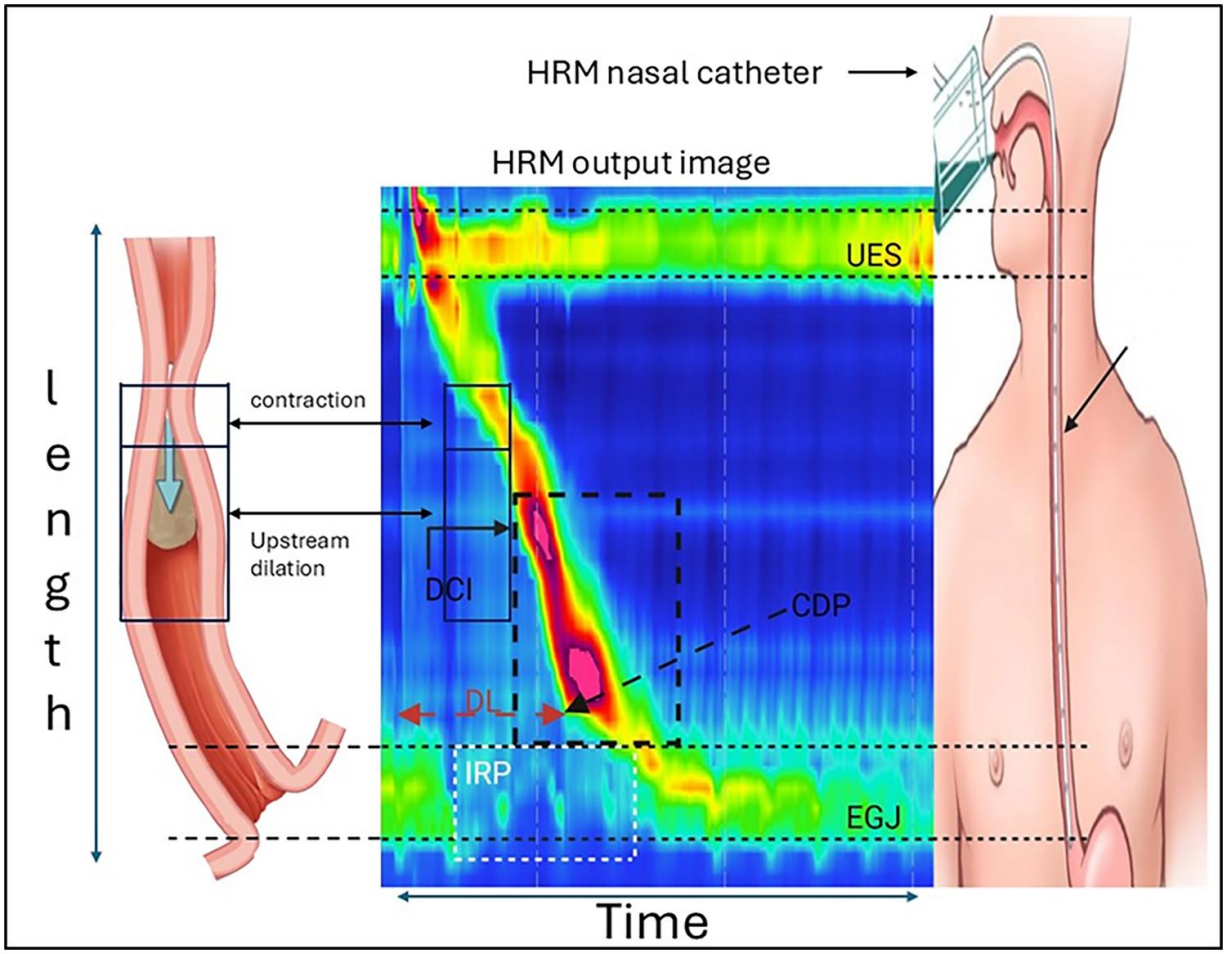
High-resolution manometry (HRM) methodology and key manometric parameters during normal esophageal peristalsis. A transnasal HRM catheter with pressure sensors at 1-cm intervals (right) records intraluminal pressure, displayed as a color pressure map (center) with esophageal position on the y-axis and time on the x-axis. Following a swallow, the upper esophageal sphincter (UES) relaxes, followed by a coordinated peristaltic wave propagating toward the esophagogastric junction (EGJ). Key metrics include distal contractile integral (DCI, mmHg·s·cm), quantifying contractile strength; distal latency (DL, seconds), measuring the interval from UES relaxation to the contractile deceleration point (CDP); and integrated relaxation pressure (IRP, mmHg), assessing EGJ relaxation. Bolus pressurization proximal to the peristaltic wave is shown schematically (left).

The HRM test begins with placement of the manometry catheter through the nares into the esophagus, followed by a 60-s quiescent phase that allows for an adaptation period, then followed by a minimum of 3 inspirations to confirm catheter placement. Subsequently, a 30-s baseline period follows to identify anatomic landmarks. Next, a sequence of 10 5-mL wet swallows is first performed in the supine position with at least 30 s between each swallow, and finally 1 multiple rapid swallow sequence is performed. The patient position is then changed, and this is followed by 5 5-mL wet swallows in the upright position, followed by 1 rapid drink challenge with 200 mL water. High-resolution manometry assesses esophageal motility patterns by measuring the amplitude of contractile events in the esophagus and its sphincters (vertical axis) in relation to time (horizontal axis). Pressure is displayed as a heat map with dark blue representing lower pressures and higher pressures colored red to purple (Figure 1).

The HRM study provides multiple metrics that help evaluate and differentiate various primary EMDs (Figure 1 and Table 1). These metrics include the integrated relaxation pressure (IRP), distal contractile integral (DCI), and distal latency (DL).

**Table 1:** Summary of high-resolution manometry metrics.

Metric	Definition	Normal values (supine)	Normal values (upright)	Clinical significance
**Integrated relaxation pressure (IRP)**	Mean EGJ pressure during the **4 s of maximal deglutitive relaxation** within a 10-s window following swallow initiation	**≤15 mmHG** (solid-state) 2.0-15.5 mmHg (5th-95th percentile) 15.5-23.5 mmHg (water-perfused)	**≤13-17 mmHg** 0-17 mmHg (5th-95th percentile) 9 mmHg (median)	**Elevated IRP (>15 mmHg) defines EGJ outflow obstruction** and is required for achalasia diagnosis
**Distal contractile integral (DCI)**	Quantifies **vigor of distal esophageal contraction**: amplitude x duration x length of contraction exceeding 20 mmHg	**450-5000 mmHg·s·cm** (Chicago Classification) 178-2828 mmHg·s·cm (recent data) 1319 mmHg·s·cm (mean)	Lower than supine median values vary by system	**DCI <100 mmHg·s·cm = failed peristalsis** **DCI <450 mmHg·s·cm = weak/ineffective swallow** **DCI >8000 mmHg·s·cm = hypercontractile**
**Contractile deceleration point (CDP)**	**Inflection point where peristaltic velocity slows** as contraction approaches the EGJ; marks transition from tubular esophagus to phrenic ampulla	Topographic landmark (not a numeric value)	Topographic landmark (not a numeric value)	**Used to calculate distal latency**; terminates the propagating peristaltic contraction
**Distal latency (DL)**	**Interval from UES relaxation to the CDP**; represents time for peristalsis to reach the distal esophagus	≥4.5 s 5.4-8.5 s (5th-95th percentile) 6.9 s (mean)	Shorter than supine 6.2-11.3 s (range varies by system)	**DL <4.5 s defines premature contractions** (distal esophageal spasm, type III achalasia)

- IRP: A measure of the relaxation pressure across the EGJ during deglutition and defined as the lowest mean pressure at the LES at 4 s during a 10-s period after a swallow. An IRP value of 15 mm Hg or higher (supine) and 12 mm Hg or higher (upright) indicates abnormal transit across the EGJ for Medtronic systems and is suggestive of incomplete LES relaxation, which may be seen in conditions such as achalasia and EGJOO.
- DCI: Measures the vigor of smooth muscle contractility in the esophagus in order to assess whether esophageal peristalsis is adequate, considering the length, duration, and amplitude of the contraction. Low-amplitude contractions are subclassified as weak (DCI of 100-450 mm mmHg·s·cm or failed peristalsis (DCI <100 mm mmHg·s·cm). In contrast, vigorous high-amplitude contractions are hypercontractile when DCI is above 8000 mm mmHg·s·cm.
- DL: Measures the timing of peristalsis based on the interval between upper esophageal sphincter relaxation and the contractile deceleration point of the peristaltic wave in the distal esophagus; the inflection point characterizes the transition from the end of esophageal peristalsis to the beginning of esophageal emptying. This helps to differentiate between premature (DL<4.5 s) and peristaltic contractions.

### Adjunctive testing to high-resolution manometry

The clinical diagnosis of EMDs relies on integrating information from patient symptoms in conjunction with adjunctive tests, particularly in cases with borderline findings on HRM or when discordant findings are noted in different positions.

A timed barium esophagram is a study used to assess emptying of barium from the esophagus at 1, 2, and 5 min following ingestion of barium. Esophagram can identify abnormalities, including esophageal stricture or neoplasm, but may also be helpful in diagnosing major motility disorders, although the overall sensitivity of esophagram for diagnosing motility disorders is between 56% and 69%.^5^^,^^6^ A retained barium column height measuring >5 cm at 1 min and measuring >2 cm at 5 min is abnormal and provides evidence for abnormal esophageal emptying.^7^

Endoluminal Functional Lumen Imaging Probe (EndoFLIP) is used to measure the distensibility and diameter of the EGJ by introducing a catheter transorally into the esophageal lumen during endoscopy.^8^ A reduction in the EGJ distensibility index and/or diameter is often seen in patients with achalasia and EGJOO.

### Disorders of esophagogastric junction outflow

**Figure 2:**
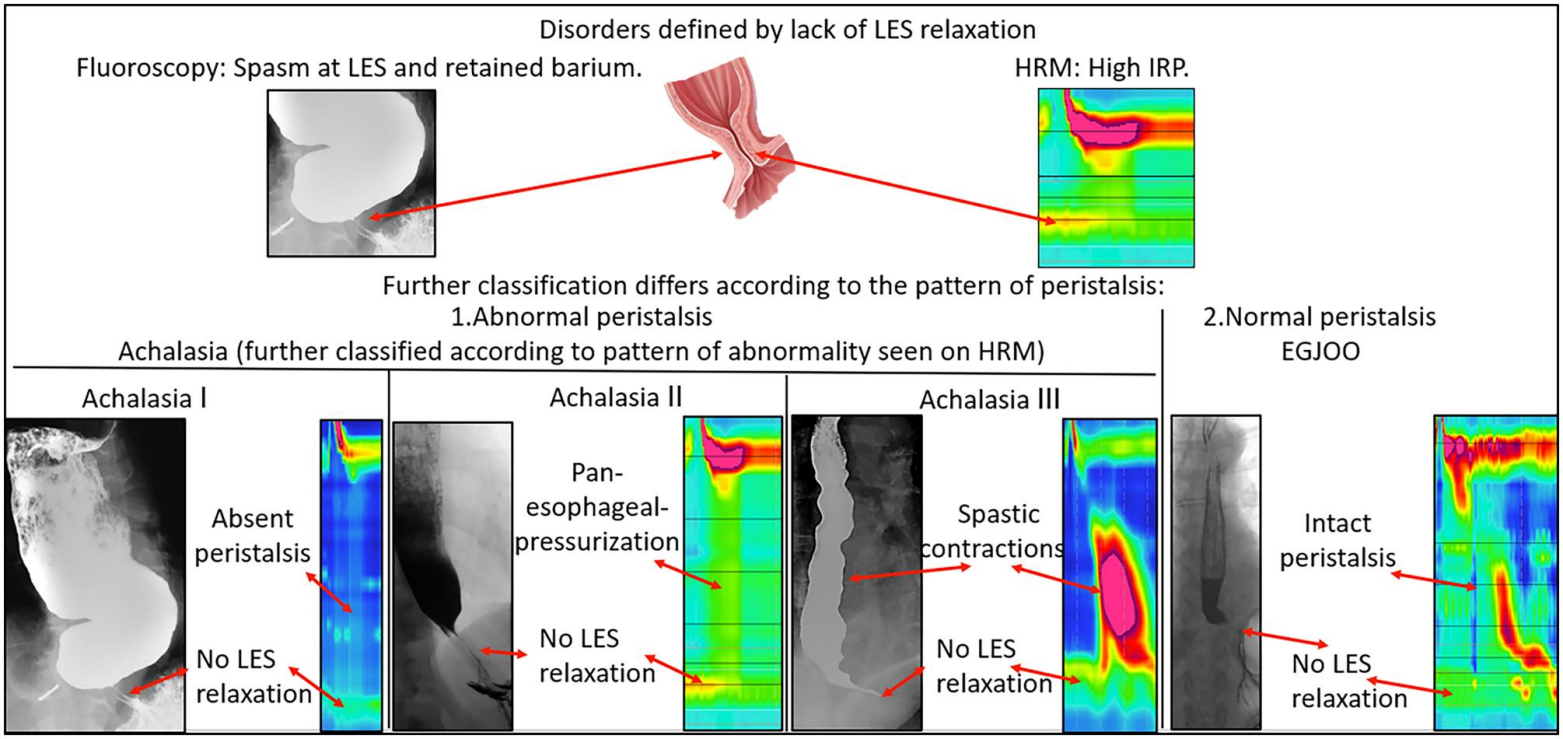
Overview of esophageal motility disorders characterized by impaired esophagogastric junction relaxation (elevated IRP >15 mmHg), with corresponding manometric and radiographic findings. Type I achalasia: absent peristalsis with no esophageal pressurization. Type II achalasia: absent peristalsis with pan-esophageal pressurization (>30 mmHg). Type III achalasia: premature contractions with shortened distal latency (<4.5 s). EGJ outflow obstruction (EGJOO): elevated IRP with preserved peristalsis.

The disorders of the esophagogastric junction include achalasia (Types I, II, and III) and EGJOO, each of which demonstrates an abnormal median IRP (Figure 2 and Table 2).

**Table 2:** Summary of disorders of esophagogastric junction outflow.

Disorder	Chicago Classification v4.0 criteria	High-resolution manometry findings	Barium esophagram findings
**Achalasia type I**	IRP >15 mmHg; 100% failed peristalsis (DCI 100 mmHg·s·cm); absent contractility	Elevated IRP; aperistalsis; minimal esophageal pressurization	Air-fluid level; bird’s beak appearance; esophageal dilation; poor emptying on TBE (>5 cm at 1 min, >2 cm at 5 min)
**Achalasia type II**	IRP >15 mmHg; 100% failed peristalsis; ≥20% premature (spastic) contractions with DCI >450 mmHg·s·cm	Elevated IRP; aperistalsis; **panesophageal pressurization**	Air-fluid level; **wave appearance**; esophageal dilation; bird’s beak; abnormal TBE
**Achalasia type III**	IRP >15 mmHg; ≥20% premature (spastic) contractions with DCI >450 mmHg·s·cm	Elevated IRP; **premature contractions** (DL 4.5 s); spastic contractions	**Rosary-bead/corkscrew appearance**; bird’s beak; esophageal dilation; abnormal TBE
**EGJ outflow obstruction**	IRP >15 mmHg; insufficient evidence of achalasia; requires supportive evidence (symptoms + abnormal TBE or provocative testing)	**Elevated IRP; preserved peristalsis**; elevated RDC-IRP (>16.7 mmHg predictive); upright IRP >12 mmHg	Abnormal retention of 13-mm tablet; TBE demonstrates poor esophageal emptying

### Achalasia

Achalasia is characterized by the absence of esophageal peristalsis and failure of LES relaxation due to the loss of inhibitory neurons in the myenteric plexus in the distal esophagus and LES.^9^ Symptoms include progressive dysphagia to solids and liquids, heartburn, regurgitation, noncardiac chest pain, and weight loss. A barium esophagram demonstrates a dilated esophagus with “bird’s beak” narrowing of the distal esophagus due to a hypertonic LES.^9^ There are 3 main subtypes of achalasia based on the predominant pattern on HRM.^10^

**Figure 3:**
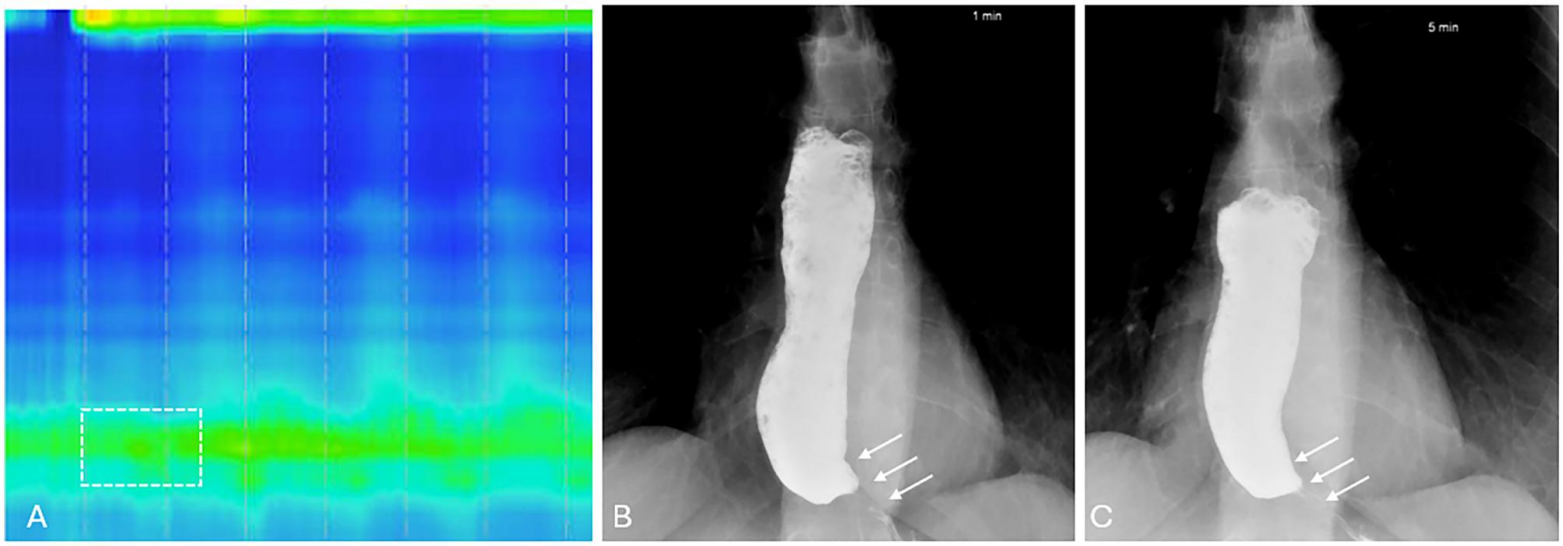
Type I achalasia in a 56-year-old female with dysphagia requiring endoscopic removal of a food bolus. (A) HRM demonstrates absent peristalsis, unmeasurable DCI, and elevated IRP of 15.8 mmHg (white dashed box). (B and C) Timed barium esophagography demonstrates a retained column of barium measuring 13.5 cm after 1 min and 11 cm after 5 min, as smooth tapered narrowing at the EGJ (white arrows).

**Figure 4:**
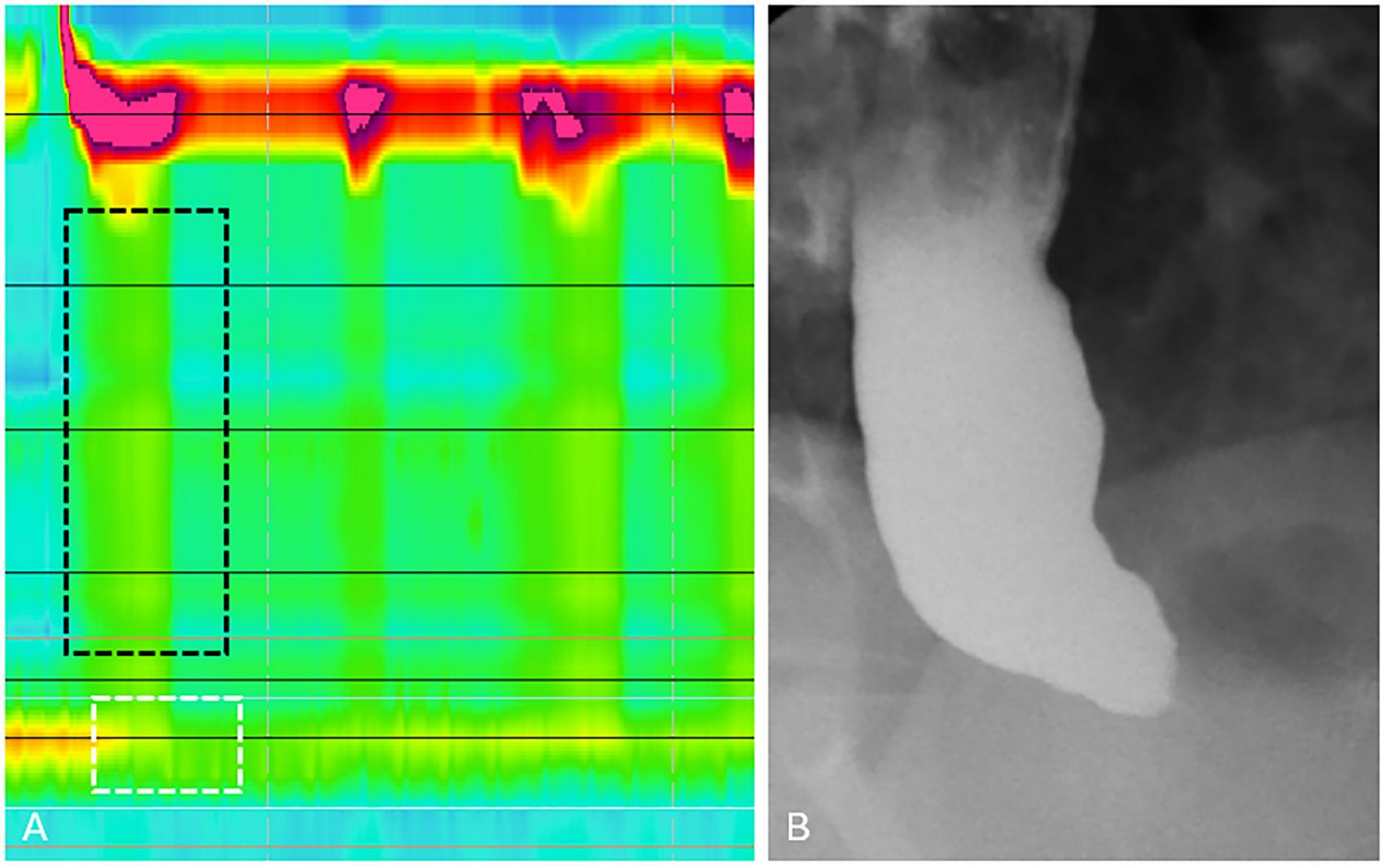
Type II achalasia in a 41-year-old male with dysphagia and regurgitation. (A) HRM demonstrates absence of peristalsis with pan-esophageal pressurization (black dashed box) and elevated IRP of 24.2 mmHg (white dashed box). (B) Barium esophagography demonstrates a retained column of barium within the esophagus and “bird’s beak” narrowing at the EGJ.

**Figure 5:**
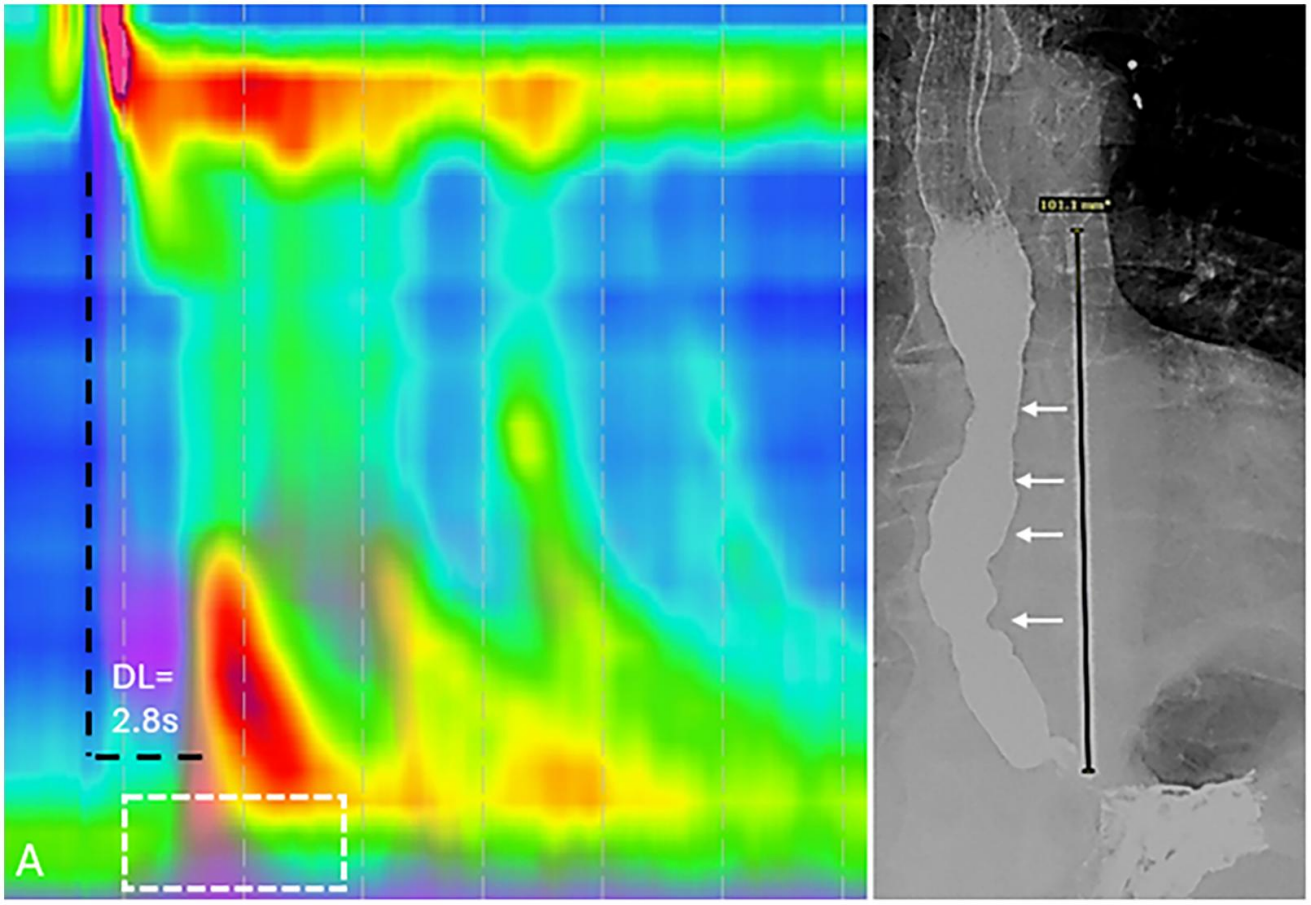
Type III achalasia in a 58-year-old male with a history of dysphagia. (A) HRM demonstrates premature contractions with DL measuring 2.8 s (black dashed lines) and elevated IRP of 36.3 mmHg (white dashed box). (B) Timed barium esophagography demonstrates a retained column of barium measuring 10.1 cm at 1 min as well as nonpropulsive tertiary contractions (white arrows).

1. Type I achalasia is characterized by an elevated median IRP across the EGJ and failure of 100% of wet swallows, thereby indicating absence of peristalsis (DCI <100 mm mmHg·s·cm) (Figure 3).
2. Type II achalasia is classified by an elevated median IRP across the EGJ, 100% failed swallows, and 20% or more of swallows demonstrating panesophageal pressurization (Figure 4).
3. Type III achalasia is characterized by an elevated median IRP across the EGJ with 20% or more of swallows that are spastic/premature (DL <4.5 s) in the setting of DCI >450 mm mmHg·s·cm (Figure 5).

A timed barium esophagram is helpful in assessing response following treatment of achalasia and demonstrates a decrease in the retained column of barium within the esophagus compared to imaging before therapy.

### Esophagogastric junction outflow obstruction (EGJOO)

EGJOO typically presents with non-mechanical obstructive symptoms, including most commonly dysphagia and non-cardiac chest pain, although there is heterogeneity in presentation and natural history. EGJOO is defined by an elevated IRP in both the upright and supine positions (>15 mm Hg in supine and >12 mm Hg in upright positions), indicating impaired EGJ relaxation, although esophageal peristalsis is maintained. A conclusive diagnosis of EGJOO, in addition to manometric findings, also requires the presence of obstructive symptoms and confirmatory testing on adjunctive tests, including timed barium esophagram or EndoFLIP.

**Figure 6:**
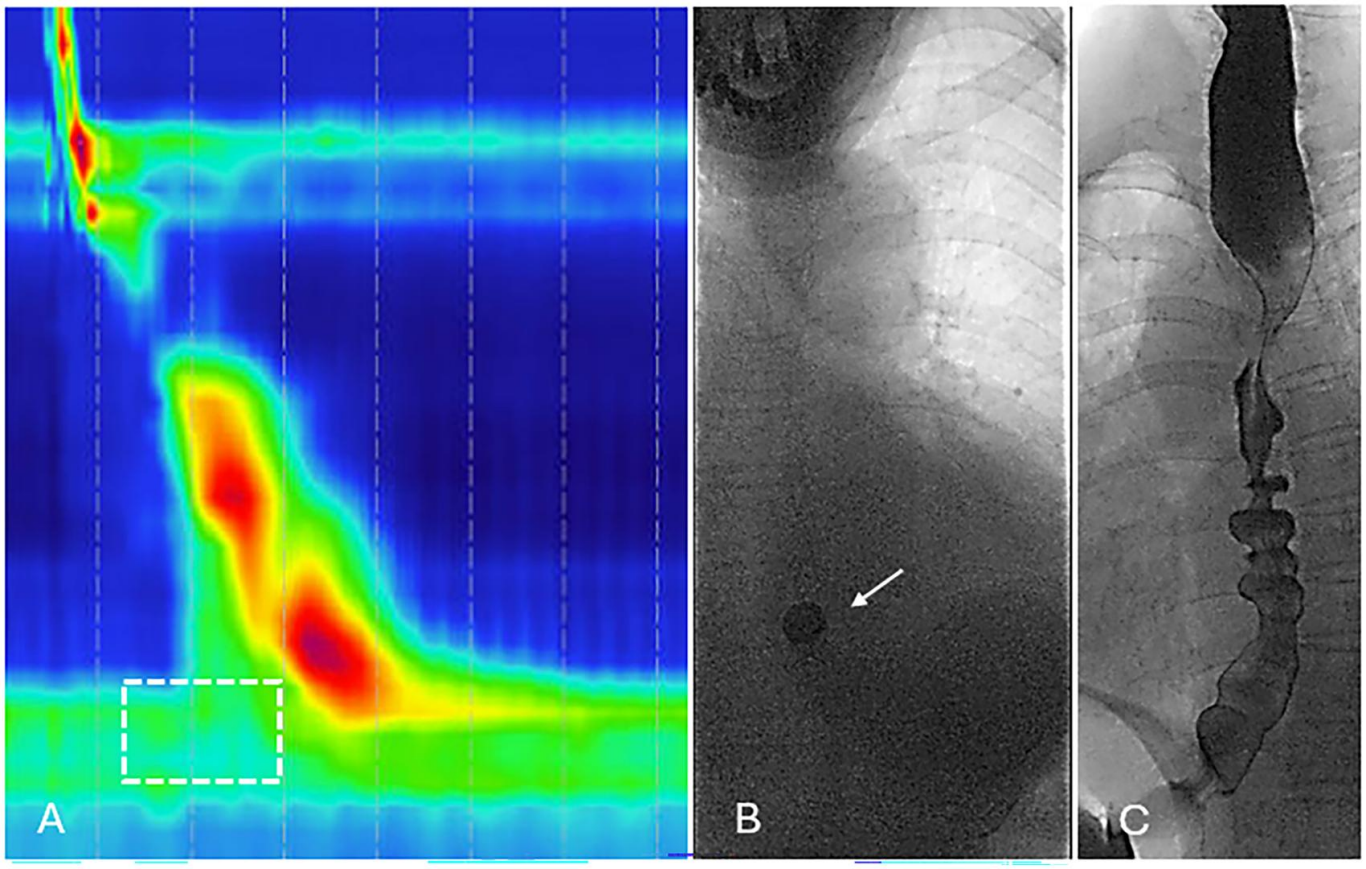
Esophagogastric junction outflow obstruction (EGJOO) in a 70-year-old female with dysphagia and gastroesophageal reflux refractory to proton pump inhibitors. (A) HRM demonstrates preserved peristalsis with normal DCI (3013 mmHg·cm·s) and DL (5.9 s), but elevated IRP measuring 23.4 mmHg (white dashed box). Barium esophagography demonstrates retention of a 13 mm tablet retention for >5 min (B, white arrow) and tertiary contractions (C).

A timed barium esophagram demonstrates poor esophageal emptying as well as retention of a 13 mm barium tablet, which can increase the diagnostic yield of EGJOO^11^ (Figure 6). Therefore, HRM and timed barium esophagram provide complementary information that are essential for accurate diagnosis and treatment planning. Additionally, findings on EndoFLIP include decreased distensibility or reduced diameter at the EGJ.^12^

### Disorders of esophageal peristalsis

**Figure 7:**
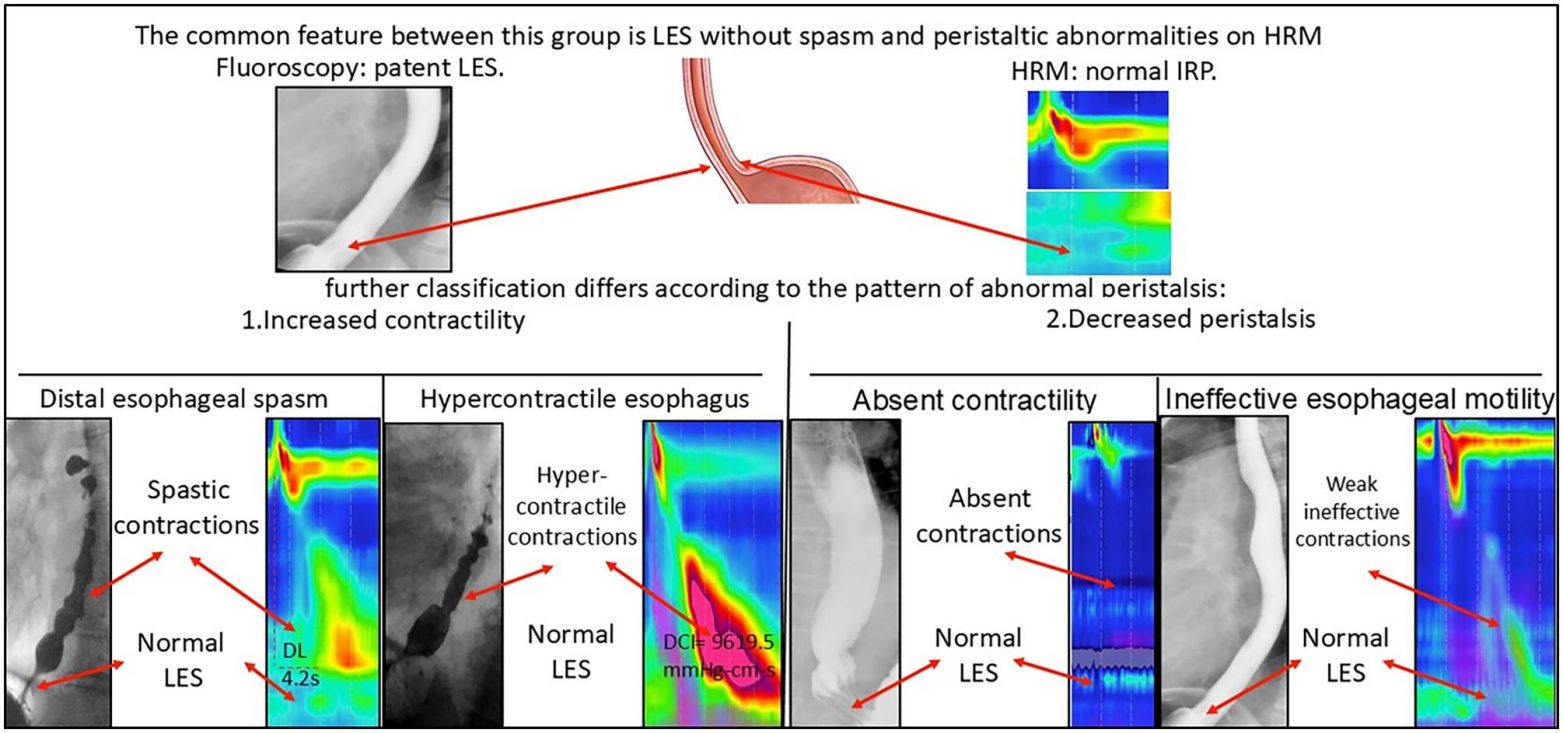
Overview of esophageal motility disorders with peristaltic abnormalities and normal LES relaxation (IRP <15 mmHg), with corresponding manometric and radiographic findings. Distal esophageal spasm (DES): Premature contractions with shortened DL (<4.5 s). Hypercontractile esophagus: excessive contractile vigor (DCI >8000 mmHg·s·cm). Absent contractility: 100% failed peristalsis (DCI <100 mmHg·s·cm). Ineffective esophageal motility: >50% weak or fragmented swallows.

Disorders of peristalsis are considered when a disorder of EGJ outflow has been excluded and are characterized by a normal median IRP but exhibit deficiencies in the contractility of the esophageal body (DCI) or DL. Furthermore, these can be divided into spastic and hypomotile disorders (Figure 7 and Table 3).

**Table 3:** Summary of disorders of esophageal peristalsis.

Disorder	Chicago Classification v4.0 criteria	High-resolution manometry findings	Barium esophagram findings
**Distal esophageal spasm**	Normal IRP; ≥20% premature contractions (DL 4.5 s) with DCI >450 mmHg·s·cm	Normal IRP; **≥20% premature contractions;** fragmented peristalsis possible	**Rosary-bead/corkscrew appearance**; non-propulsive tertiary contractions
**Hypercontractile esophagus**	Normal IRP; ≥20% swallows with DCI >8000 mmHg·s·cm	Normal IRP; **DCI >8000 mmHg·s·cm in ≥20 swallows**; multipeaked contractions; normal DL; may have hypertensive LES	Often **normal**; may show vigorous peristalsis or tertiary contractions
**Ineffective esophageal motility**	>70% ineffective swallows and/or ≥50-70% ineffective with supportive evidence	Normal IRP; **>70% ineffective swallows** (weak: DCI 100-450 mmHg·s·cm; fragmented: breaks >5 cm; failed: DCI 100 mmHg·s·cm); absent contraction reserve on MRS	**Absent/weak peristalsis;** delayed transit in the supine position
**Absent contractility**	100% failed peristalsis (DCI 100 mmHg·s·cm); normal IRP	Normal IRP; **100% failed peristalsis**; complete aperistalsis of smooth muscle esophagus	**Absent/weak peristalsis;** delayed esophageal emptying

### Distal esophageal spasm (DES)

**Figure 8:**
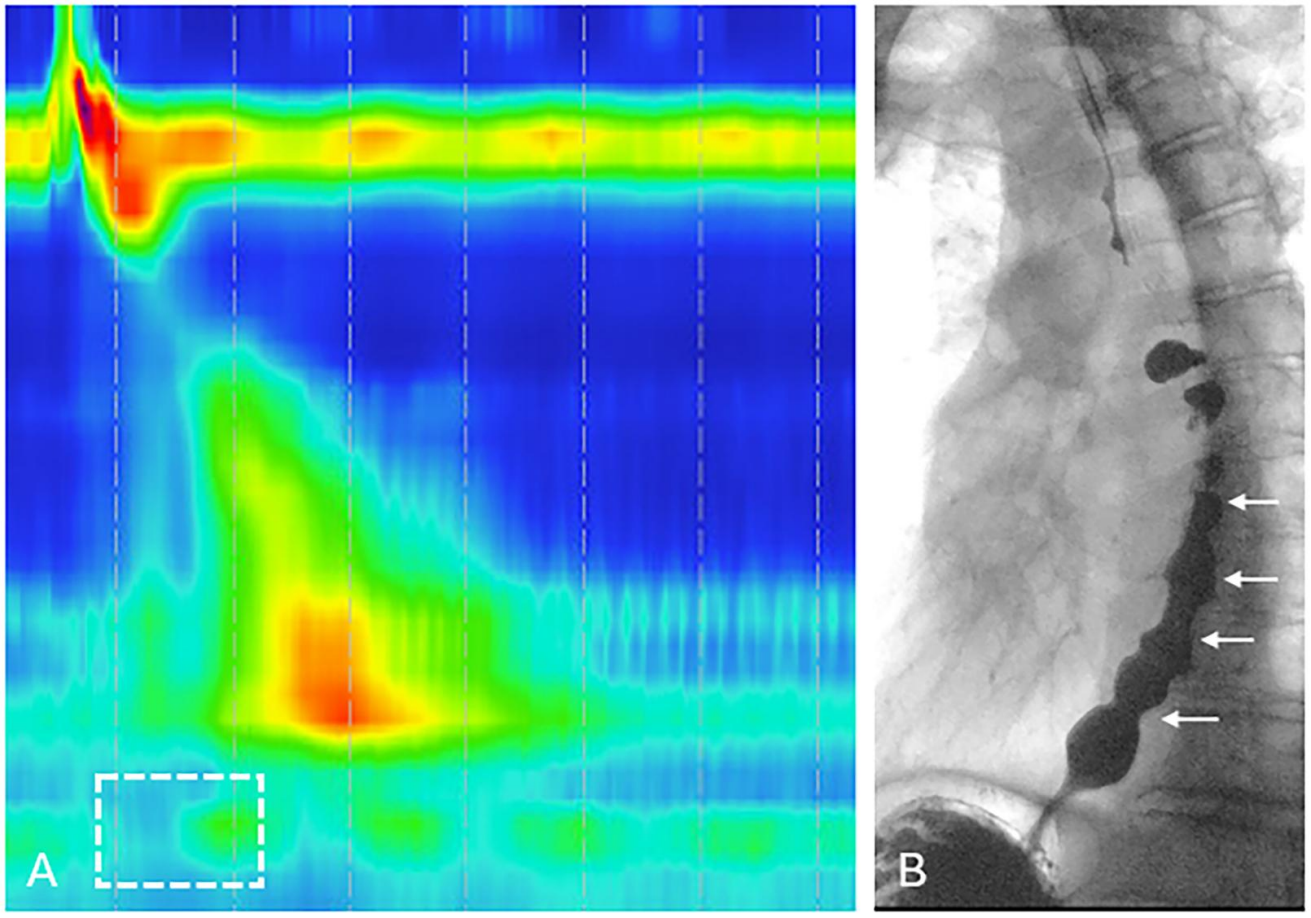
Distal esophageal spasm in a 69-year-old male with dysphagia and regurgitation. (A) HRM demonstrates premature contractions with shortened DL measuring 4.2 s and normal IRP of 4.8 mmHg (white dashed box). (B) Barium esophagography demonstrates the “rosary-bead” appearance of the esophagus (white arrows), with passage of barium into the stomach.

DES describes an abnormal esophageal motility pattern characterized by spastic or premature contractions in the distal esophagus, which is defined as an esophageal contraction with DL <4.5 s, in the setting of DCI >450 mm mmHg·s·cm. The diagnosis requires clinically relevant symptoms such as dysphagia and non-cardiac chest pain in conjunction with manometric findings of at least 20% premature contractions in the setting of normal lower esophageal sphincter relaxation.^13^ Barium esophagography may demonstrate a “corkscrew” or “rosary bead” esophagus associated with DES, although this is relatively uncommon (Figure 8).^14^^,^^15^ Additionally, patients may have beak-like narrowing of the distal esophagus on barium esophagography due to LES dysfunction.^16^

### Hypercontractile esophagus

**Figure 9:**
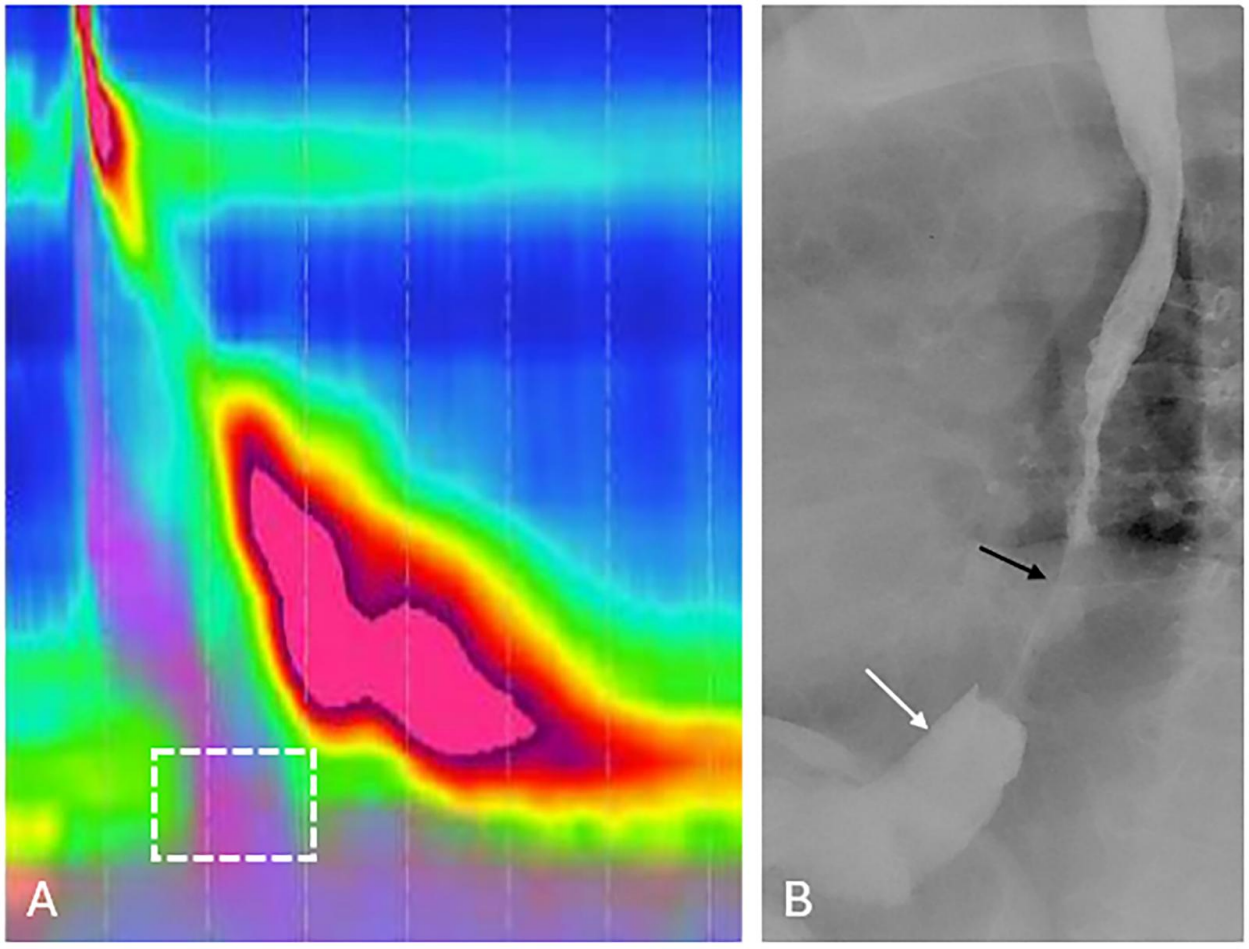
Hypercontractile esophagus in a 71-year-old female presenting with dysphagia to solids and liquids. (A) HRM demonstrates elevated DCI exceeding 8000 mmHg·cm·s (maximum 9619.5 mmHg·cm·s) and normal IRP of 12.5 mmHg (white dashed box). (B) Barium esophagography demonstrates vigorous contractions in the distal esophagus resulting in luminal narrowing (black arrow) as well as a small sliding hiatal hernia (white arrow).

Hypercontractile esophagus is diagnosed on HRM when ≥20% of swallows demonstrate hypercontractile peristalsis with a DCI >8000 mm mmHg·s·cm in the context of normal LES relaxation. The CCv4.0 describes that a conclusive diagnosis requires both manometric findings and clinically relevant symptoms of dysphagia, non-cardiac chest pain, or regurgitation. Supportive findings on HRM include multiple rapid swallows or a rapid drink challenge in order to help improve diagnostic confidence.^17^ Findings on barium esophagography may be normal despite the increased contraction amplitude (Figure 9).

### Absent contractility

**Figure 10:**
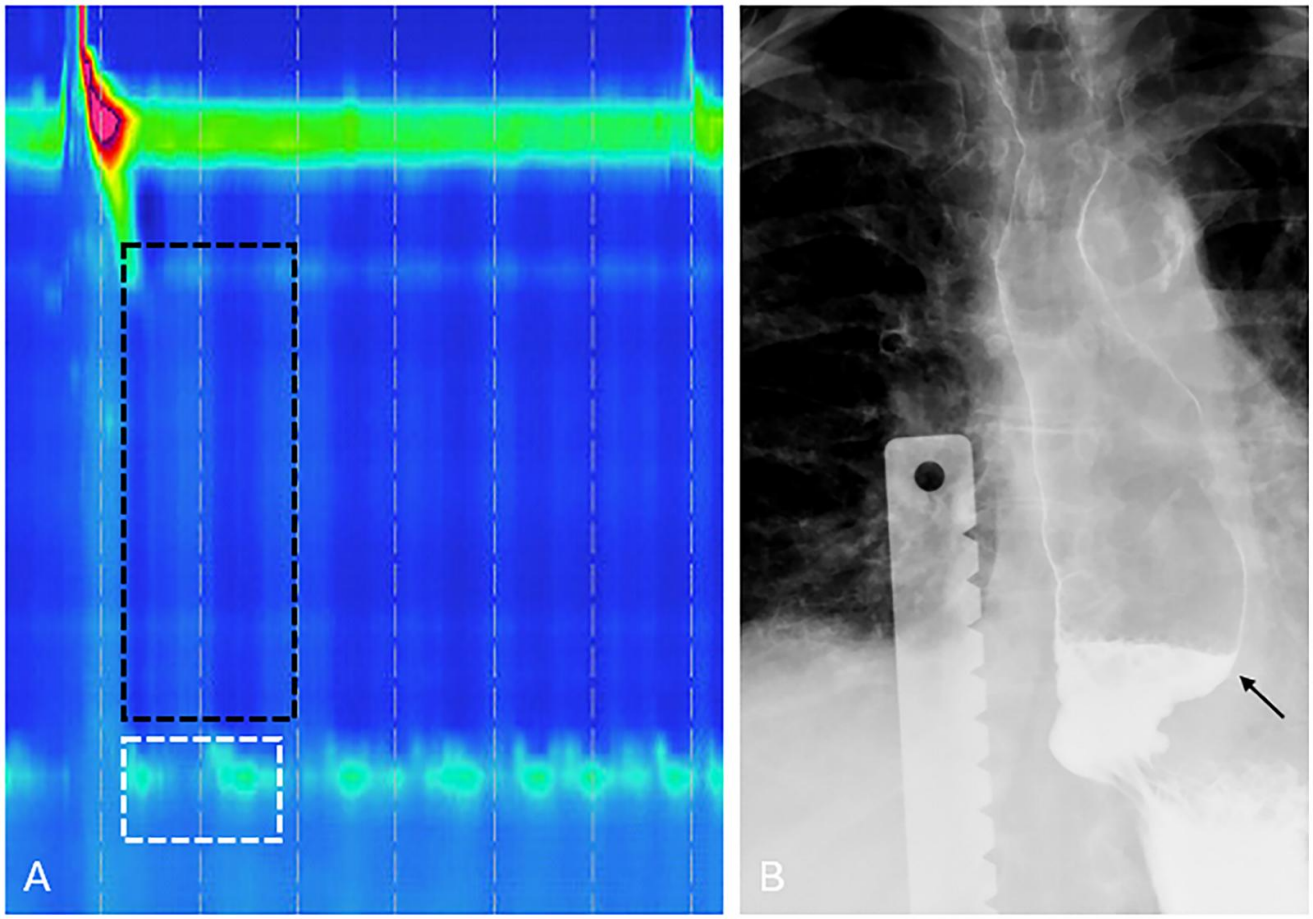
Absent contractility in a 79-year-old female with dysphagia and scleroderma. (A) HRM demonstrates complete absence of peristalsis (DCI <100 mmHg·s·cm; black dashed box) and normal IRP of -1.0 mmHg (white dashed box). (B) Barium esophagography demonstrates esophageal dilatation (black arrow) with associated absence of esophageal peristalsis noted on fluoroscopic examination.

Absent contractility is a hypomotility disorder defined as 100% failed peristalsis (DCI <100 mm mmHg·s·cm) along with a normal median IRP in the supine and upright positions. As both type I achalasia and absent contractility demonstrate 100% failed swallows, the differentiating feature is based on normal IRP (absent contractility) vs elevated IRP (type I achalasia). In cases of borderline median IRP values, additional testing with a timed barium esophagram with a tablet can be useful in order to rule out retention of barium in the upright position, as is commonly seen with type I achalasia.^18^ Barium esophagography may show delayed bolus transit or abnormal emptying but often appears normal or nonspecific and does not consistently demonstrate the absence of peristaltic contractions (Figure 10). Patients with absent contractility have a higher prevalence in patients with connective tissue disorders, including scleroderma.^19^

### Ineffective esophageal motility

**Figure 11:**
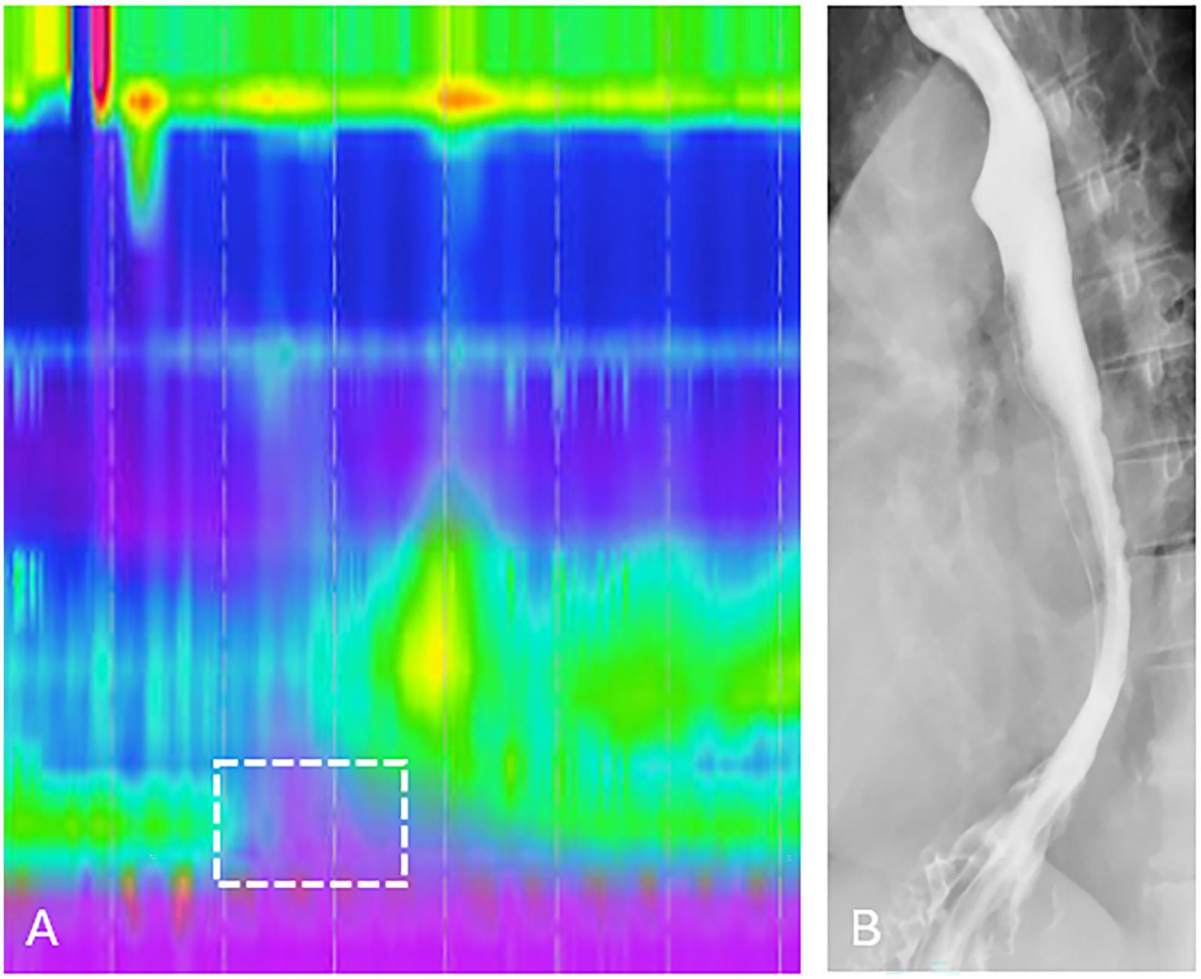
Ineffective esophageal motility in a 78-year-old female with dysphagia and regurgitation. (A) HRM demonstrates >50% ineffective swallows (approximately 90%), DCI between 100 and 450 mmHg·cm·s and normal IRP of 14.1 mmHg (white dashed box). (B) Barium esophagography demonstrates fragmented peristaltic waves and incomplete clearance of barium from the esophagus.

Ineffective esophageal motility is characterized by diminished esophageal peristaltic vigor with weak, absent, and/or fragmented peristalsis. The diagnosis of IEM requires more than 70% ineffective swallows (ie. weak contractions with DCI ≥100 mmHg·s·cm and <450 mmHg·s·cm; failed peristalsis with DCI <100 mmHg·s·cm; or a fragmented swallow) and/or ≥50% failed peristalsis (DCI <100 mmHg·s·cm). Ineffective esophageal motility has been found in up to 10%-17% of asymptomatic patients, and symptoms include reflux, chronic cough, and chest pain.^20^ Supportive evidence for the diagnosis of IEM includes poor bolus transit in the recumbent position during barium esophagram (Figure 11).

### Secondary esophageal motility disorders

#### Connective tissue disorders

Scleroderma and Sjogren’s Syndrome are the most common connective tissue disorders associated with esophageal dysmotility and resulting in symptoms of dysphagia. Esophageal involvement occurs in up to 90% of patients with scleroderma, and manometric findings include decreased LES pressure as well as absent contractility.^21^ Findings on barium esophagography correlate with manometry and demonstrate impaired or absent peristalsis and impaired function of the LES related to atrophy and fibrosis of the smooth muscle in the distal two-thirds of the esophagus. Additional findings include dilatation of the esophagus, hiatal hernia and patulous gastroesophageal junction; therefore patients may also have gastroesophageal reflux (Figure 10). Similar manometric patterns can also occur in mixed connective tissue disease, rheumatoid arthritis, and systemic lupus erythematosus.

#### Opioid-induced esophageal dysfunction

**Figure 12:**
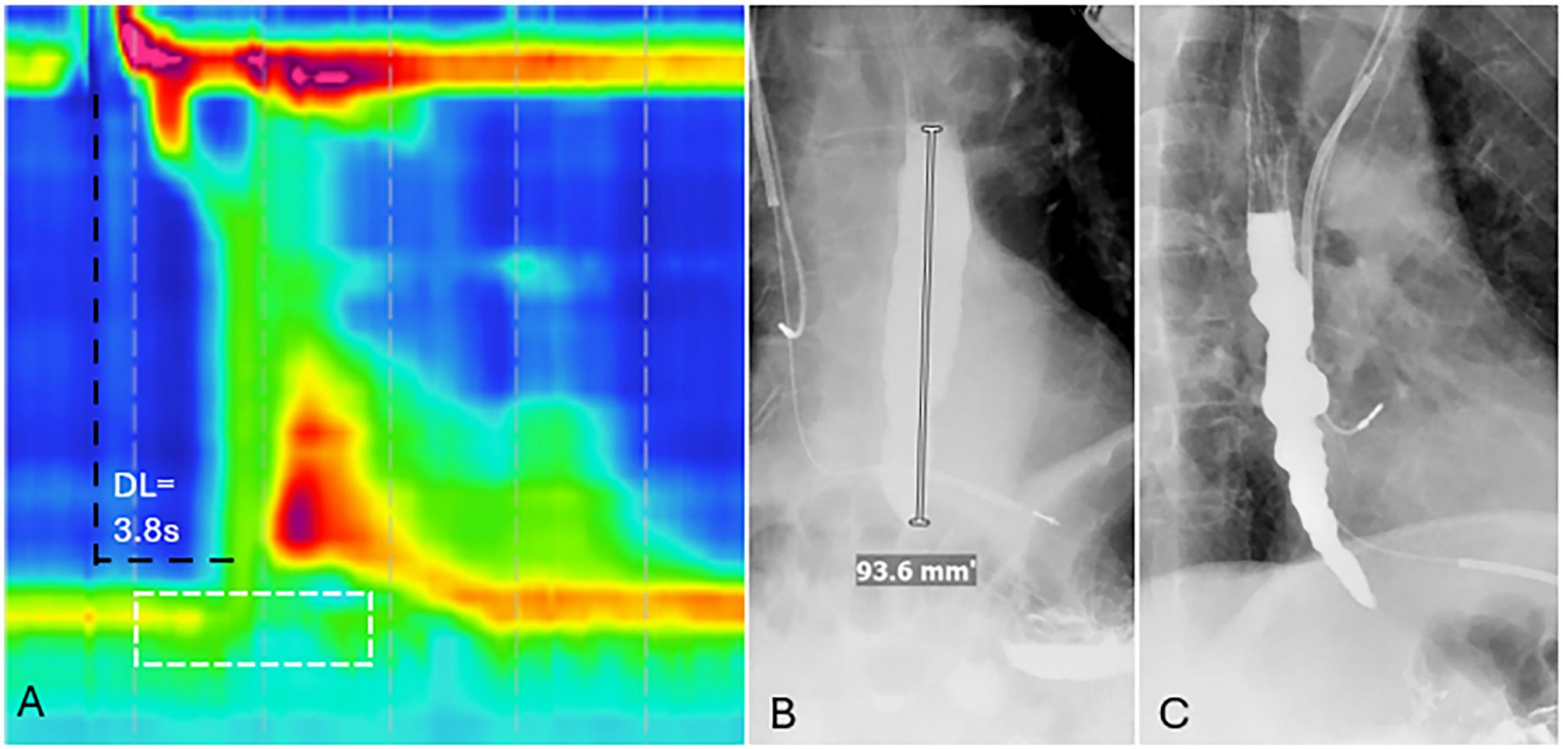
Opioid-induced type III achalasia in a 68-year-old male with dysphagia on chronic opioid therapy for back pain. (A) HRM demonstrates premature contractions with a shortened DL of 3.6 s (black dashed lines) and an elevated IRP of 24 mmHg (white dashed box). Timed barium esophagography demonstrates LES spasm with a retained column of barium measuring 9.3 cm at 5 min (B) and tertiary contractions (C) consistent with opioid-induced dysmotility.

Kraichely *et al.* documented the effects of chronic opioid use on esophageal motility using conventional manometry and demonstrated that 12/15 patients had abnormal motility defined by at least 30% non-peristaltic contractions, and abnormalities included high-amplitude contractions and incomplete LES relaxation.^22^ Patients may present with dysphagia as well as symptoms of gastroesophageal reflux and chest pain. Opioid-induced esophageal dysfunction is a clinical diagnosis based on symptoms, opioid use, and manometric evidence of DES, EGJOO, or type III achalasia. The best way to discern between opioid-induced vs. idiopathic dysmotility is to repeat esophageal manometry after cessation of opioids (Figure 12).

#### Esophageal dysmotility following antireflux and bariatric surgery

**Figure 13:**
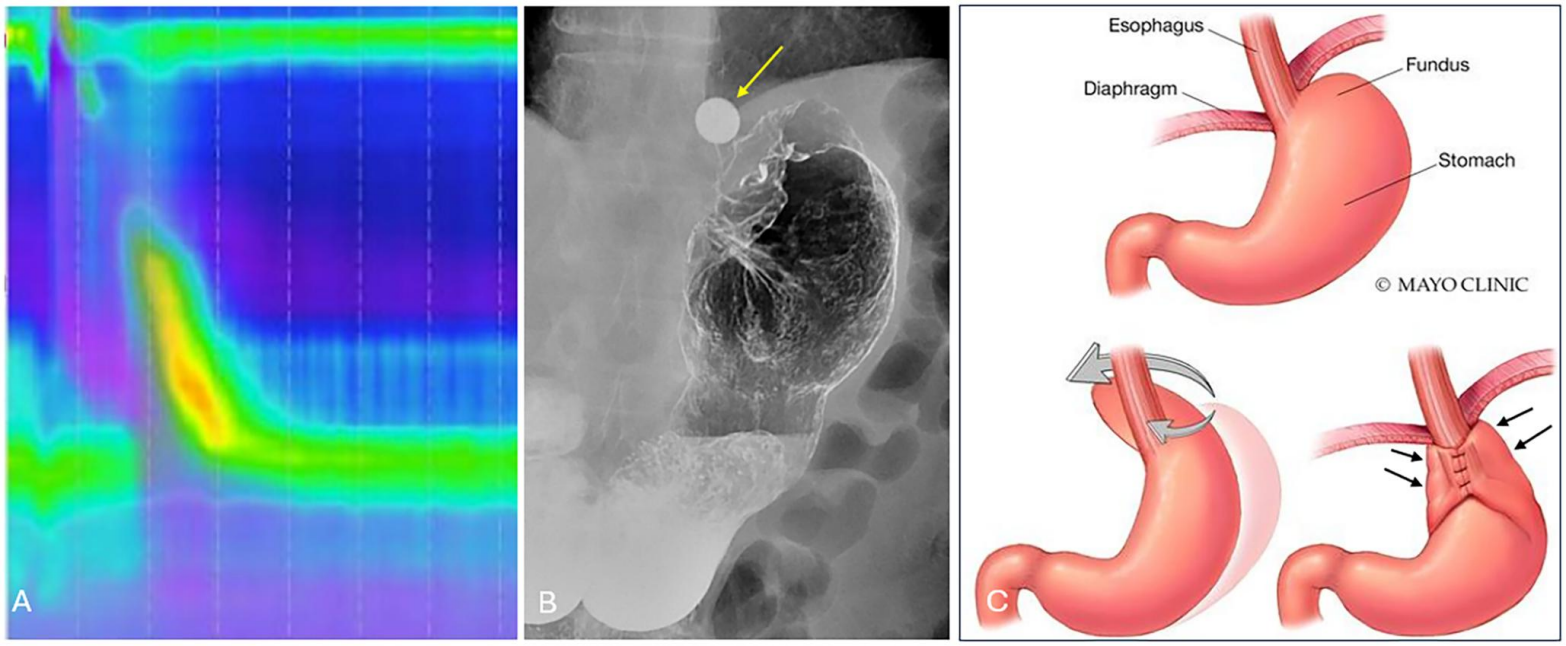
A 60-year-old male presenting with dysphagia 3 months following Nissen fundoplication. (A) HRM demonstrates preserved peristalsis with an elevated IRP of 17.9 mmHg. (B) Barium esophagography demonstrates retention of the 13 mm tablet retention for >20 min. (C) Nissen fundoplication surgical anatomy; the gastric fundus is mobilized and wrapped 360° posteriorly around the distal esophagus (gray arrows), creating a neo-sphincter mechanism at the esophagogastric junction (EGJ). The wrap is secured with interrupted sutures anteriorly, reinforcing lower esophageal sphincter (LES) competence and preventing gastroesophageal reflux. The fundoplication is anchored below the diaphragmatic hiatus to maintain the LES in an intra-abdominal position (black arrows).

Esophageal dysmotility after antireflux surgery has been described in up to 7% of patients after laparoscopic Nissen fundoplication, and manometry may demonstrate abnormal EGJ morphology or EGJ outlet obstruction.^23^^,^^24^ EndoFLIP can assess for reduced EGJ distensibility, which may be associated with dysphagia symptoms. Barium esophagography demonstrates delayed esophageal emptying and retention of the 13 mm barium tablet, as well as additional complications such as wrap herniation or slippage (Figure 13).

**Figure 14:**
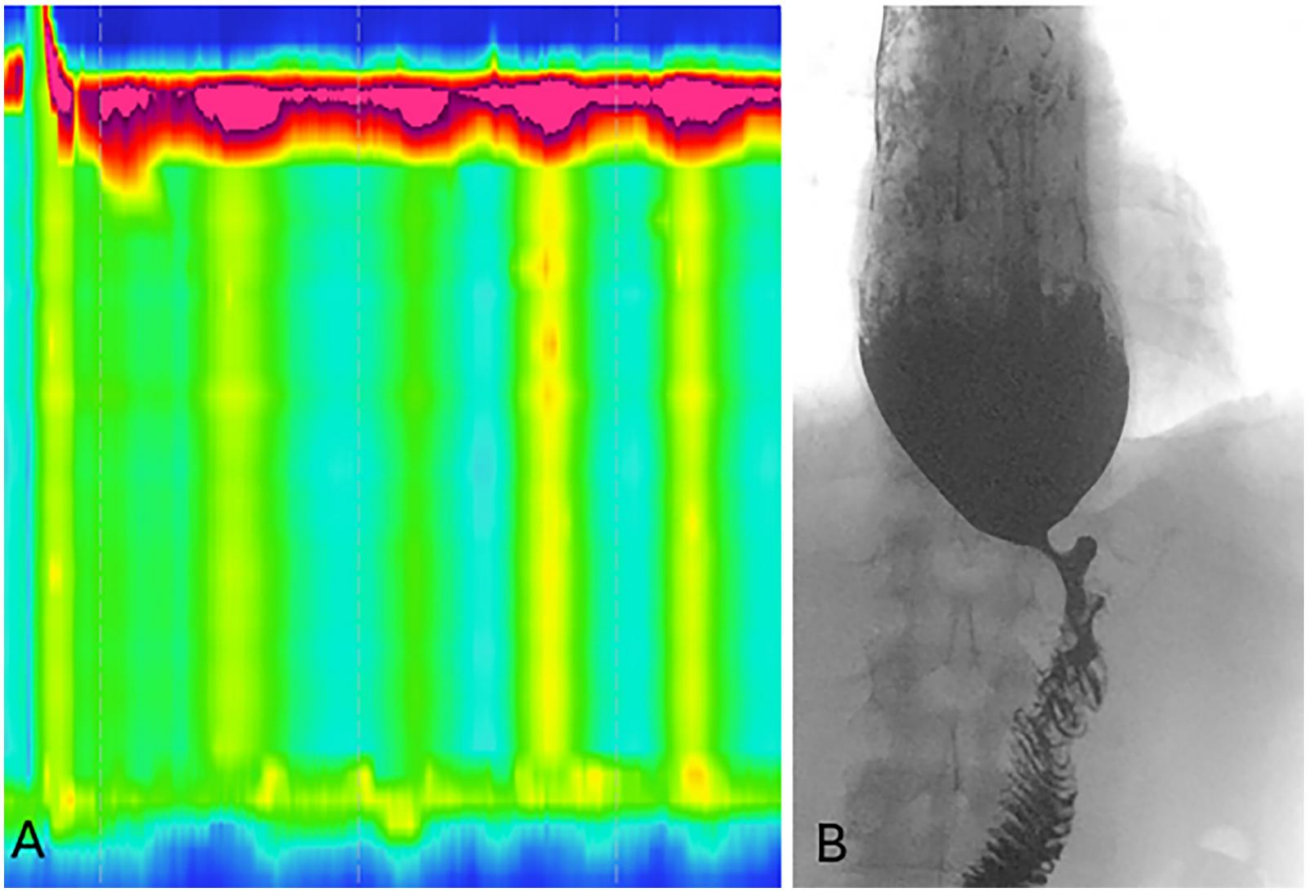
A 45-year-old male with a history of Roux-en-Y gastric bypass presenting with dysphagia, regurgitation, and weight loss. (A) HRM demonstrates pan-esophageal pressurization and elevated IRP (>15 mmHg), consistent with vagal injury-induced achalasia. (B) Timed barium esophagography demonstrates LES spasm resulting in esophageal dilation and a retained column of barium measuring 4.8 cm after 5 min. The patient subsequently required peroral endoscopic myotomy for treatment.

Postoperative dysphagia following bariatric surgery has a prevalence of 13.7% at mean of 3.9 years after surgery and was reported in 51% of patients after laparoscopic sleeve gastrectomy and 46% of patients after Roux-en-Y gastric bypass.^25^ Furthermore, a manometric pattern consistent with achalasia was found in 7.2% of postsurgical patients versus 0% preoperatively, and an additional 5.2% developed post-obesity surgery esophageal dysfunction (POSED), characterized by aperistalsis and increased intragastric pressure. Barium esophagography may be useful to assess for anastomotic stricture or other anatomic abnormalities (Figure 14).

## Conclusion

Esophageal motility disorders represent a spectrum of disorders with overlapping symptoms, most commonly resulting in dysphagia. CCv4.0 is a classification system for the evaluation and diagnosis of motility disorders using metrics from HRM. However, the data obtained from HRM remain part of a complementary diagnostic approach that integrates clinical symptoms, endoscopic findings, and radiographic imaging. Barium esophagography, particularly timed barium esophagram with the use of a tablet, provides critical adjunctive information, particularly in cases with inconclusive manometric findings. Radiologists play a crucial role in the diagnostic evaluation of EMDs by recognizing characteristic patterns on barium esophagography that correlate with specific manometric diagnoses described by gastroenterologists. Therefore, understanding the correlation between radiographic findings and HRM metrics allows radiologists to provide clinically meaningful interpretations that help guide further diagnostic workup and therapeutic decision-making.
